# Records of Human Deaths from Echinococcosis in Brazil, 1995–2016

**DOI:** 10.3390/vetsci9080436

**Published:** 2022-08-16

**Authors:** Michael Laurence Zini Lise, Jo Widdicombe, Claudia Ribeiro Zini Lise, Stefan Vilges de Oliveira, Eduardo Pacheco de Caldas, Mahbod Entezami, Joaquín M. Prada, Nilton Ghiotti, Rosângela Rodrigues e Silva, Katherina A. Vizcaychipi, Victor Del Rio Vilas

**Affiliations:** 1 Secretaria de Vigilância em Saúde, Ministério da Saúde do Brasil, Brasília 70300-904, Brazil; 2 School of Veterinary Medicine, University of Surrey, Guildford GU2 7AL, UK; 3 Secretaria de Assistência à Saúde, Ministério da Saúde do Brasil, Brasília 70300-904, Brazil; 4 Federal University of Acre, Rio Branco 69920-900, Brazil; 5 General Surgeon at Hospital de Clínicas do Acre, Rio Branco 3222-4326/3306, Brazil; 6 Laboratório de Helmintos Parasitos de Vertebrados, Instituto Oswaldo Cruz, Rio de Janeiro 21040-360, Brazil; 7 Instituto Nacional de Enfermedades Infecciosas e Instituto Nacional de Medicina Tropical-ANLIS “Dr. Carlos G. Malbran”, Ministerio de Salud de la Nación, Buenos Aires C1282AFF, Argentina; 8 Center for Universal Health, St. James’s Square, London SW1Y 4LE, UK

**Keywords:** mortality, Brazil, Morbidity, hospital information system, mortality information system

## Abstract

**Simple Summary:**

Echinococcosis is a zoonotic disease relevant to public health in many countries. The disease is present in Brazil; however, it is often underreported due to the lack of mandatory notification of cases across all Brazilian states. The records of two national databases were accessed during the period of 1995–2016 to describe the registered cases and deaths from echinococcosis in the country. Demographic, epidemiological, and health care data related to the occurrence of disease, and deaths attributed to echinococcosis are described. During the study period, 7955 hospitalizations were recorded due to echinococcosis, with 185 deaths. In a second database recording just mortality, a further 113 deaths were documented. Deaths were observed in every state of Brazil. When comparing between states, there was great variability in mortality rates, possibly indicating differences in the quality of health care received by patients and reinforcing the need to expand the compulsory notification of the disease across the country.

**Abstract:**

Echinococcosis is a zoonotic disease relevant to public health in many countries, on all continents except Antarctica. The objective of the study is to describe the registered cases and mortality from echinococcosis in Brazil, from 1995 to 2016. The records of two national databases, the Hospital Information System (HIS) and the Mortality Information System (MIS), were accessed during the period of 1995–2016. Demographic, epidemiological, and health care data related to the occurrence of disease and deaths attributed to echinococcosis in Brazil are described. The results showed that 7955 records of hospitalizations were documented in the HIS, during the study period, with 185 deaths from echinococcosis, and 113 records of deaths were documented in the MIS Deaths in every state of Brazil in the period. When comparing between states, the HIS showed great variability in mortality rates, possibly indicating heterogeneity in diagnosis and in the quality of health care received by patients. Less severe cases that do not require specialized care are not recorded by the information systems, thus the true burden of the disease could be underrepresented in the country. A change in the coding of disease records in the HIS in the late 1990s, (the integration of echinococcosis cases with other pathologies), led to the loss of specificity of the records. The records showed a wide geographic distribution of deaths from echinococcosis, reinforcing the need to expand the notification of the disease in Brazil. Currently, notification of cases is compulsory in the state of Rio Grande do Sul.

## 1. Introduction

Echinococcosis, also known as Hydatidosis, is a zoonotic disease caused by parasites of the genus *Echinococcus* spp. (Phylum Platyhelminthes, Class Cestoda, Order Cyclophyllidea, Family Taeniidae). Whilst some Echinococcus spp. are transmitted in cycles that predominantly involve domestic dogs and livestock, the parasite is adapted to a predator–prey relationship (carnivores–herbivores and omnivores) and therefore can also be transmitted in complex multihost systems affecting both domestic and wild mammals [1,2]. Wildlife transmission may be primary or secondary, however, spillover events and transmission cycles in exclusively wildlife species are poorly documented [1]. The parasite is predominantly found in rural or wild areas, and in South America has been identified in bush dogs (*Speothos venaticus*), lowland pacas (ground-dwelling, herbivorous rodents, *Cuniculus paca*), as well as more commonly in dogs and the ungulate intermediate hosts associated with the local farming practices [3,4].

Accidental human infections occur when eggs expelled in the feces of their carnivore definitive hosts are dispersed in the environment and are inadvertently ingested by humans. This may be via contaminated water or food, or when there is direct contact with feces found in animal hair [5,6,7,8,9]. After ingestion, the embryonated eggs hatch within the small intestines and release the larval form of the parasite known as oncospheres [7]. Oncospheres subsequently penetrate through the intestinal mucosa and spread to the liver and other body organs, whereupon larval development starts and cystic structures form.

The development period of the cyst varies from months to years depending on the speed of development and location of the cyst [9]. Due to the zoonotic nature of the disease, people living in close proximity to their livestock or in remote rural locations, such as in pastoral farming communities, are disproportionately affected. Anthropogenic hunting practices in rural areas may also contribute to disease transmission. For example, in the state of Acre in Brazil, whereupon bushmeat is consumed regularly, domestic hunting dogs may be fed discarded offal containing hydatid cysts, and subsequently become infected with CE [4]. Consequently, this may facilitate human infections [5,10]. Control of the disease relies upon interrupting transmission through interventions in the definitive and intermediate hosts, such as dog and sheep deworming treatments and sheep vaccination [11]. However, preventative measures that prevent *Echinococcus* egg ingestion may help to reduce the disease burden in humans [12].

The clinical presentation of the disease is highly variable and is related to cyst location and the organ affected. Patients can remain asymptomatic for long periods of time, for example, regarding inpatients with hepatic cysts, about 75% of infections do not present with clinical signs and symptoms for more than 10 years [13]. Morbidity and mortality is more likely to occur if the cyst affects vital organs such as the lungs, liver, and spleen, causing complications such as vascular strangulation and biliary obstruction [14,15,16].

Currently, only *Echinococcus granulosus* causes cystic echinococcosis (CE). *Echinococcus multilocularis* is the causative agent of alveolar echinococcosis (AE), and *Echinococcus vogeli* and *Echinococcus oligarthrus* cause polycystic neotropical echinococcosis (PNS) and unicystic echinococcosis (UES) respectively. *E. granulosus* is considered the most relevant species for humans. With a global distribution, CE is the most frequently incurred and causes both a medical and economic burden, but all are important zoonoses that present major health problems in several regions of the world, causing considerable socioeconomic losses [17,18,19,20,21,22,23].

In 2001, CE was considered a neglected disease by the World Health Organisation (WHO) [17] and since 2013 it has been the target of enhanced effort towards its control and elimination [24,25]. The WHO officially made the commitment to promote initiatives that support the elimination of echinococcosis, and most recently, CE was included as one of 20 neglected tropical diseases in the WHO’s latest roadmap (2021–2030) to end their neglect and attain the United Nations Sustainable Development Goals [26]. A part of this project is to enhance the capacity and effectiveness of national control and prevention programs, and the development of priority activities in pilot areas of endemic regions. According to estimates from 2006, CE results in 1009,661 (95% CI, 862,119–1175,654) global DALYs lost (disability-adjusted life years), with an annual cost of USD763 million per year worldwide, which means a higher impact than other infectious diseases highlighted more by the media and the medical community [18].

In South America, the countries most affected by CE are Argentina, Uruguay, Chile, Peru, and Brazil, with a mortality rate in the region between 0.5% to 4% in registered surgical cases [6]. PNS, with a documented mortality of 15.5–29% is present in tropical and subtropical regions of South and Central America [4,19,23].

In Brazil, three etiological agents have been identified: *E. granulosus*; *E. vogeli*, and *E. oligarthrus* [7,19,27]; however, definitive identification of the exact agent involved in the death of patients is still considered difficult. This is due to variable epidemiological analyses and diagnostic capacity available (due to both staff capability and facilities available) to be able to identify the causative agent in the laboratory, as well as a lack of formal identification of abdominal masses or neoplasms, especially when the cysts develop in unusual and atypical places [23,28,29].

Currently in Brazil the morbidity and mortality of human cases is underestimated. Experts believe that the published cases represent only a small proportion of the true number of cases and that the infection is underdiagnosed and underreported by public health agencies [19,30]. The disease is still poorly associated with *E. oligarthrus* because of the small number of human cases reported in endemic regions [6].

In Brazil, two information systems make it possible to record information on echinococcosis deaths: the Hospital Information System (HIS) and the Mortality Information System (MIS). The HIS identifies hospitalization records according to demographic data, cause, length of stay, geographic distribution and costs, and has a managerial function within the Brazilian Health System [31,32]. The MIS was developed with the purpose of gathering quantitative and qualitative data on deaths, which is used by the management and for epidemiological surveillance.

There are no nationwide studies documenting deaths from echinococcosis in Brazil. Previous studies are case reports of individual patients that do not allow evaluation of the overall magnitude of deaths related to CE. Thus, this study aims to describe the registered cases and mortality from echinococcosis in Brazil, from 1995 to 2016, using both the hospital information system and mortality information system (HIS and MIS respectively).

## 2. Materials and Methods

A retrospective descriptive study was conducted using data on hospital admissions that led to CE-related deaths. These data were recorded in the HIS from January 1995 to December 2016, and deaths recorded in the MIS were from January 1999 to December 2016. The MIS does not provide data with the same time interval as the HIS, (with a difference of 4 years), making it impossible to fully cross-reference the historical data series.

For the extraction of data in the two information systems, we identified records that had the International Classification of Diseases ICD-09 for the years 1995 to 2000, and ICD-10 B67 as a base cause, and the years 2000 to 2016 as subcategories, as listed below.

B67.0 *Echinococcus granulosus* liver infection

B67.1 *Echinococcus* granulosus lung infection

B67.2 Echinococcus granulosus bone infection

B67.3 *Echinococcus granulosus* infections, other and of multiple sites

B67.4 Unspecified *Echinococcus granulosus* infection

B67.5 Echinococcus multilocularis liver infection

B67.6 *Echinococcus multilocularis* infections, other and of multiple sites

B67.7 Infection unspecified by *Echinococcus multilocularis*

B67.8 *Echinococcus* infection of liver, unspecified

B67.9 *Echinococcus* infections, other and unspecified

From these records, demographic data were evaluated according to the municipality, federal unit and region of residence of the patient; sex; race/color (indigenous, white, brown, black); stratified age group (<1, 1 to 4, 5 to 9, 10 to 14, 15 to 19, 20 to 24, 25 to 29, 30 to 34, 35 to 39, 40 to 44, 45 to 49, 50 to 54, 55 to 59, 60 to 64, 65 to 69, 70 to 74, 75 to 79 and > 80 years); education (years of study); and year of registration. The following epidemiological data were also evaluated: characterization of the etiological agent (*E. granulosus*, *E. multilocularis* and *Echinococcus* spp.); location of the cystic lesion (hepatic, pulmonary, bone and others (e.g., brain and bone)); and diagnostic test used to identify the cause of death. Blank values are shown, with the exception of records that did not contain information on the municipality of residence. Those records were not included in the analysis because it was impossible to evaluate the geographical distribution of cases without this information.

The results were presented separately, according to their database of origin. It was not possible to correlate the information of the patients records between the respective databases due to unavailability of the nominal records of patients. However, we compared the time and location of the records between the two data sources to safeguard against duplication.

The Hospital Mortality Rate (HMR) calculated was defined by the Ministry of Health as the ratio between the number of deaths and the number of approved hospital admission authorizations, computed as hospitalizations for echinococcosis, in the period, multiplied by 100 inhabitants.

The mortality rate (MR) was calculated using the number of hospitalizations for echinococcosis in the same period and locality. Due to the lack of the mandatory reporting of cases, patients often go undiagnosed until they are hospitalized, hence the true number of cases is unknown.

To investigate whether there was a significant difference between the populations recorded in the MIS and HIS data sets, a Wilcoxon Rank test was performed. Associations were deemed significant when *p* < 0.05. Using SIM data, indirectly standardized mortality ratios (SMR) were calculated for race and schooling levels in each federal unit. Population data was taken from the Instituto Brasileiro de Geografia e Estatística [33]. For the variables in which population data were available (sex, race, civil status), the odds ratio (OR) and the 95% confidence intervals between groups were calculated. The study was conducted with secondary data, available to the public, with no personal or identifying information accessed or held by the study team.

## 3. Results

Of the 7955 hospital admissions in the HIS, there were 185 deaths registered as *Echinococcus* spp. during the study period. We excluded 269 hospital admissions and 6 deaths by *Echinococcus* spp. that did not provide the patients place of residence. In the years in which deaths occurred, the number of records ranged from 1 in 2002 to 68 in 1995, with the average number of deaths as 16.8 per year. No deaths were registered in the HIS between 2003 and 2010.

**Figure 1:**
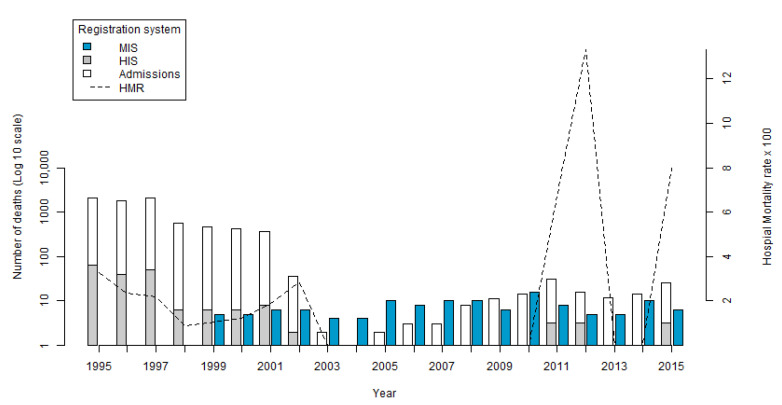
Distribution of deaths, hospital admissions, and hospital mortality recorded in HIS and MIS, by year, in Brazil, between 1995 and 2016 (expressed as Log10).

The HMR presented an average of 3.97 throughout the period. Starting from 3.15 in 1995, the lowest value of 0.86 was in 1998, and the highest value of 13.33 was in 2012 (Figure 1) (created using R 3.6.0-Core Team (2019) [34]). In contrast to the number of hospitalizations that was reduced, an increase in the HMR was seen at the end of the period.

In the MIS, 112 records of deaths by *Echinococcus* spp. were identified during the period from 1999 to 2016. The highest number of records occurred in the year 2010 with 15 deaths (13.3%) and the lowest in the years 2003 and 2004 with three deaths (2.7%) (Figure 1). The average number of deaths per year was 6.3.

A total of 1287 (23%) municipalities where hospitalized cases resided were identified, of which 106 (8.2%) had deaths registered in the HIS. There were hospitalizations in all Federal Units (FUs), but deaths, although recorded in all regions, were concentrated in 18 FUs. The FU state of Bahia registered 59 deaths (31.9%), followed by São Paulo with 23 deaths (12.4%), Rio de Janeiro with 19 deaths (10.3%), and Rio Grande do Sul with 17 deaths (9.2%). The Northeast region recorded 98 deaths (53.0%), followed by the Southeast with 51 deaths (27.6%), the South with 27 deaths (14.6%), the North with 8 deaths (4.3%), and the Centre-West with o1ne death (0.5%). The highest number of deaths in the HIS occurred in the municipality of Salvador with 25 deaths (13.5%) recorded, followed by Maceió with 11 deaths (5.9%), and Recife and Rio de Janeiro with 9 deaths (4.9%) recorded in each.

**Figure 2:**
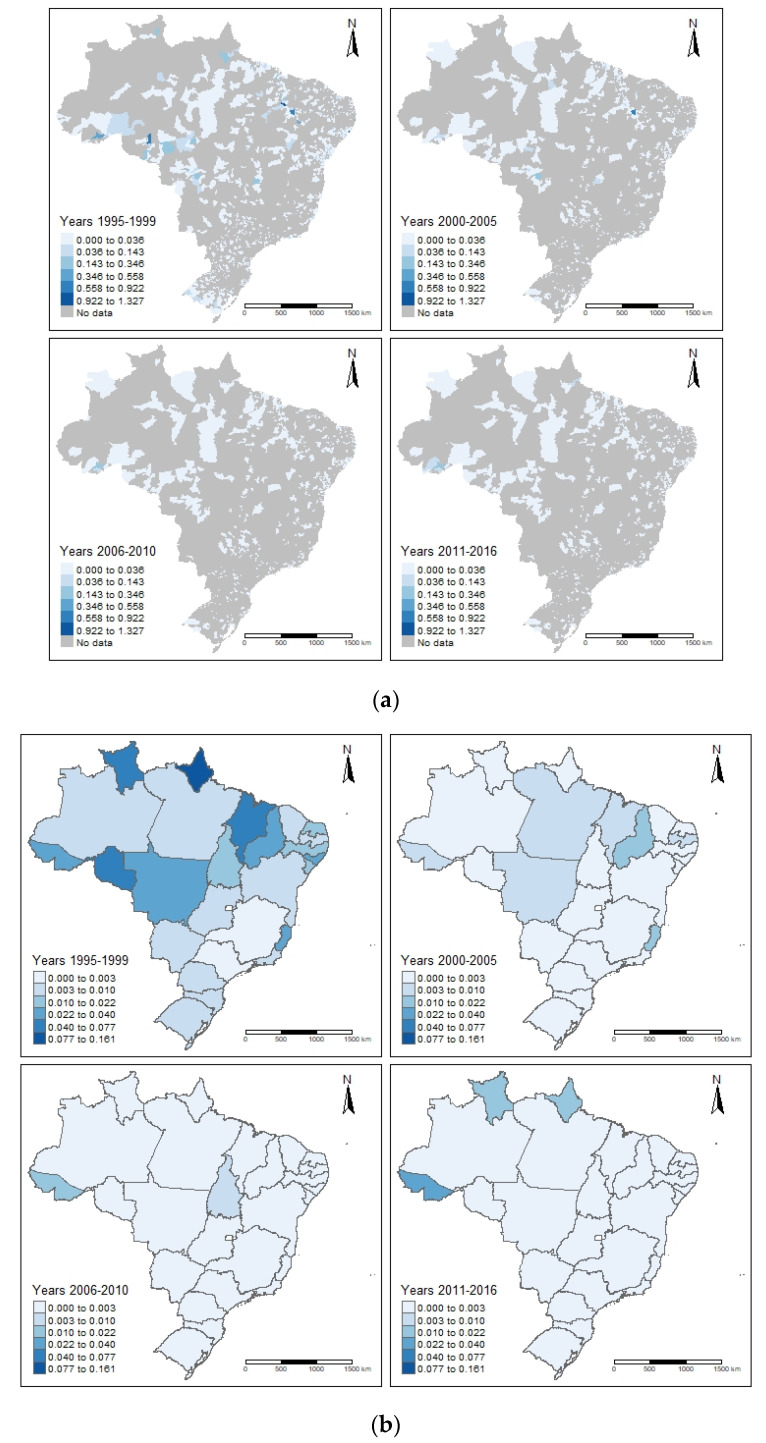
(**a**) Distribution of deaths recorded in MIS and HIS, by municipality, in Brazil, from 1999 to 2016 (calculated as the average number of deaths/year per 100,000 population). (**b**) Distribution of deaths recorded in MIS and HIS, by state level (FU), in Brazil, from 1999 to 2016 (calculated as the average number of deaths/year per 100,000 population).

In the MIS, 71 municipalities distributed across 20 Federal units were identified as the recorded place of death. The state with the highest number of residential municipalities was Rio Grande do Sul with 26 (36.2%), followed by São Paulo with 7 (9.86%), Acre with 6 (8.45%), and Pará with 4 (5.63%). Alagoas and Piauí registered three (4.23%) municipalities each. The highest concentration of municipalities with records of deaths registered in the MIS occurred in the Southern region was 30 (42.25%) and Northern region 15 (21.13%). The Northeast, Southeast, and Central-west regions registered 11 (15.49%), 9 (12.68%), and 6 (8.45%), respectively. The overlap of municipalities with at least one death recorded in both systems, was 13 (12.3%). Deaths shown by both municipality level (Figure 2a) and state level (Figure 2b) are shown below. (Figures created using R package tmap: Thematic Maps in R [35]).

**Figure 3:**
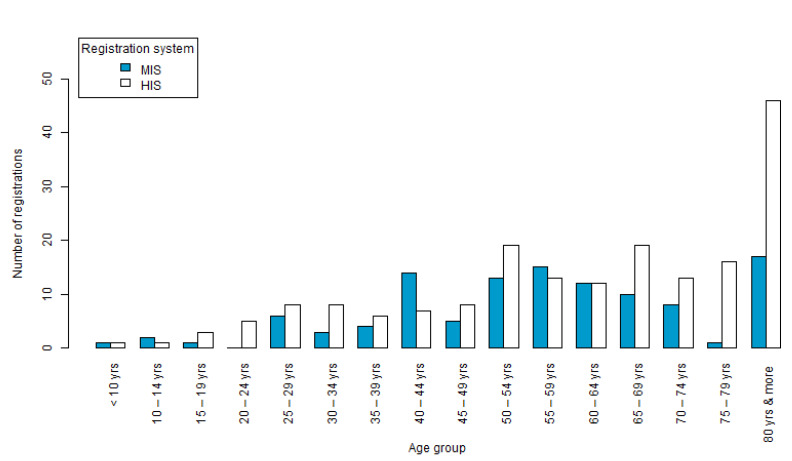
Deaths recorded in HIS and MIS, in Brazil, from 1995 to 2016, by age.

The age range of deaths in the HIS varied from less than 1 year (*n* = 1; 0.5%) to more than 80 years old (*n* = 48; 25.1%). The most deaths were recorded in patients between the ages of 45 and 74 years (*n* = 87; 45.6%). In the MIS, the age of death ranged from 7 years old to 92 years old with at least one recorded death in each age category. The median age of death in the MIS was 56.5 years old. The age groups with the most deaths recorded were 46–55 and 56–65 years, with 23 deaths in each, totaling 46 (40.71%) of the total recorded deaths. There were three records (2.65%) of deaths in children less than 15 years old, and 18 deaths documented in the 75–79 year old category and over (15.93%) (Figure 3).

Males were the most prevalent sex of those persons who had died in both systems, with 108 deaths (56.54%) in the HIS and 69 deaths (61.61%) in the MIS. Ethnicity characteristics can only be evaluated between 2008 and 2016, in the HIS, when six deaths were registered. Four deaths (66.67%) were of people with brown skin, followed by one death (16.67%) of both white skinned and unidentified race. In the MIS, between 1999 and 2016, deaths in white skinned people were more prevalent with 61 (54.5%) of the records, followed by brown skinned people with 31 records (27.7%), and black skin documented in 9 of the records (8.0%). There was one (0.9%) death of an indigenous person, and 10 deaths in which the race of the person was not documented (8.9%).

There was no significant difference between the age and sex of persons recorded in the HIS and MIS. Odds ratio showed that males were more likely to have died from CE than females (OR 1.67, CI 1.14–2.45, *p* value 0.007). Civil status also affected disease outcome with married persons (OR 2.28, CI 1.45–3.59, *p* < 0.001) and widowers (OR 2.54, CI 1.45–4.44, *p* < 0.001) more likely to have died from CE than single people.

Adjusting for race, significantly higher than expected mortality ratios were seen in the regions of Acre (SMR 41.61, CI 20.66–69.84, *p* < 0.001) and Pará (SMR 3.89, CI 1.76–6.85, *p* < 0.001). In comparison, Minas Gerais (SMR 0.10, CI 0.00–0.41, *p* < 0.001) and Rio de Janeiro (SMR 0.12, CI 0.00–0.4, *p* value 0.003) showed significantly lower than expected people dying. Acre (SMR 42.29, CI 23.04 -67.34, *p* < 0.001), Pará (SMR 3.4, CI 1.62 -5.84, *p* < 0.001), and Rio Grande do Sul (SMR 8.43, CI 6.28 -10.9, *p* < 0.001) showed higher than expected mortality ratios, and Minas Gerais significantly less (SMR 0.0094, CI 0.00–0.37, *p* < 0.001).

Schooling was measured in years of study and was only available in the MIS. Most people had between 1 and 3 years of education (30, 26.79%), followed by no years of study (15, 13.39%). Patients with more than 8 years of study were identified in 10 records (8.93%), and years of schooling not documented was present in 47 records (41.96%). SMR by schooling showed a similar distribution to that of race.

The mortality rate (MR) and mortality rate per 100,000 inhabitants, by Federal unit, can be seen in Table 1. Deaths registered in the MIS, as coded by the International Classification of Disease-10 causes of death, and mortality rate per 100,000 inhabitants, are presented in Table 2.

The distribution of lesions, as given in the MIS by etiological agent and cyst location, revealed the liver as the main site of cyst location (*n* = 29, 25.66%), followed by the lung (*n* = 11, 9.73%). The location of cysts was not recorded in 53 patient records (46.9%) (Table 3).

MIS data showed that further diagnostic tests were performed in the laboratory to identify the cause of death in 43 (38.39%) of the deaths. In 25 patients (22.32%), the cause of death was confirmed by surgery, and 3 (2.65%) underwent full necropsy by specialist physicians.

By looking at the year of deaths, location of death, age, and race, it was possible to establish that cases in the SIH did not duplicate that of the SIM with the exception of four cases possibly registered in both systems. With all personal data available exhausted we were unable to confirm whether these four cases were duplicates in the data documented in both recording systems.

## 4. Discussion

One of the main characteristics of the two information systems used is that they identify only the cases that resulted in death. Thus, there is likely an underestimation of the overall cases of echinococcosis reported in Brazil.

With the update from ICD-9 to ICD-10 in the early 2000s, and the change in the way hospital admissions were registered, there was a decrease in the number of registrations in the HIS, as observed in the data for historical hospital admissions.

Another important difference between the two systems is the temporal distribution of deaths. Following commencement of both recording system, the largest number of deaths in the HIS occurred in 2001, with seven deaths (24.1%). In the same year, five deaths (4.4%) were recorded in the MIS. In 2010, there were no deaths in the HIS, however, in the MIS there were 15 (13.3%) records. Possible explanations for these differences are that the deaths did not occur in a hospital environment and were instead in the patient’s own home. This may be due to the difficulty of hospitalization for patients (potentially due to access of healthcare or due to cost). Failure to identify the disease in time to receive timely hospital treatment may also have been a contributing factor. At the hospital level, there may have been inadequacies in the registration of the cause of deaths.

The variations in hospital mortality rates identified in the HIS reflect the decrease in the number of hospitalizations identified in the study. No hospital admissions were recorded during the period 2003–2010. After this time, admissions increased again, reaching a peak in 2012. During the same period, two deaths occurred and the HMR reached its peak. Another possible cause of this increase would be the hospitalization of very severe cases with advanced disease presentations, and limited treatment options, subsequently resulting in death.

It was not possible to nominally assess the deaths or even identify a patients place of exposure due to the chronicity of the disease, which may have lasted for several years. Even so, the findings corroborate studies carried out by other authors who identified the existence of different species of *Echinococcus*, both in humans and in animal hosts, distributed in various geographical regions of Brazil [28,36,37,38].

The higher number of deaths recorded in the state of Rio Grande do Sul in the MIS may be a consequence of the local human epidemiological surveillance efforts in the region since the early 1990s. This resulted in the training of the health network, and the diagnosis of the disease reinforced with compulsory notification of human echinococcosis cases in Rio Grande do Sul in 2010, as per the publication of Ordinance 210 in the region. Rio Grande do Sul is the only state in the country where disease notification is compulsory and occurs systematically, and also the only state which presented deaths throughout the analyzed historical time period.

The large number of existing records in Rio Grande do Sul showed high rates of deaths in the Northwest and Northeast regions of Acre and Pará, respectively. These are areas that are known to harbor different strains of *Echinococcus* such as *E. granulosus* in the South and *E. vogeli* in the North [28,36,37,38,39]. Records of deaths and hospitalizations in the Northeast region of Brazil need to be better evaluated, since this area is not considered endemic for the parasite.

Conversely, the occurrence of deaths in states which are known to be endemic for echinococcosis may indicate difficulty in identifying the cause of death or lack of knowledge of the agent in the region on the part of health agents. The opposite may also be true, since states where no human cases of echinococcosis were identified reported the existence of the agent’s circulation.

Other factors such as not linking the location of infection to the municipality of residence of the deaths may affect the recording of cases and our understanding of the geographical distribution. In the past few decades there has been an exodus of people moving from rural locations to the large metropolitan regions in Brazil.

This, alongside the long period of incubation of the parasite, makes mapping of the cases difficult as the address given at the time of a person’s hospitalization or death may not be representative of where the person became infected, or spent the majority of their life prior to living in a more urbanized area [36,40]. Given the zoonotic nature of the disease, it would be useful to elucidate if infected patients had previously lived in close contact with dogs, livestock species, and wildlife in which the disease normally cycles. This would add further information to the risk of disease, and animal husbandry and anthropogenic factors that may contribute to disease transmission to humans in regions of Brazil. The authors assumed this as a limitation in the study.

The age of the deaths is consistent with the chronicity of the disease since it affects mainly people 15 years old and onwards. This information also supports a study conducted in Argentina. However, the average age of infection in Brazil was 56.5 years compared to 48.2 years in the Argentinian study [40]. In a study conducted in Uruguay between the years 2008 and 2011, only two cases were under 20 years of age among 55 studied [41].

In a review study that assessed 131 published works, the mean age was 45.0 years old, also below the values identified in this study [21]. This once again reinforces the need for improvement in the early diagnosis of the infection, providing timely care and treatment with the aim of reducing mortality rates. The occurrence of the disease in age groups under 10 years old is considered a good indicator of the maintenance of the parasite, since it reveals the presence of the infectious agent and risk factors in the environment in which they live [41].

The odds of men dying from CE was 1.67 more than women. CE. This can be attributed to increased exposure to manual labor and agricultural work, activities traditionally carried out by men. Married persons were also identified as more likely to die from CE than single people (OR 2.28), however, these results may vary according to age. In a study by Siqueira et al. (2003), 52.3% of males were identified, while male deaths accounted for 61.95% in the present study. Conversely, in a study carried out in Europe, it was observed that most cases occur in women [42].

The systems allow the recording of events involving *E. multilocularis*, an agent which has not yet been identified in Brazil. It is possible that a death recorded as due to *E. multilocularis* was an error. *E. granulosus* in most cases is unicystic, however, the identification of more than one cyst in the same patient may have raised suspicions of *E. multilocularis* as the causative agent. Therefore, a multicystic finding could generate a recording error, especially given the difficulty of isolating the agent in biological material and being able to support the diagnosis via laboratory methods.

Of the identified deaths, a portion of patients previously underwent surgical procedures as a treatment method, demonstrating the chronicity and possible clinical evolution of the disease. Considering the expense of surgical procedures and hospitalization of patients to the Brazilian public service, studies should evaluate the burden of the disease and the economic impact generated by the disease to health systems [18,32].

The performance of autopsies for diagnostic confirmation and measurement of the quality of medical care is a practice that is in decline [43,44]. In this study, although the basic cause has been registered as echinococcosis, in more than half of the deaths no confirmatory autopsy exam was performed. This may be due to technical and operational difficulties in the Brazilian death verification services. In 2006, the Ministry of Health instituted the National Network of physicians from the autopsy service (Serviços de verificação de óbitos, SVO physicians, Brazil) to reorganize the existing services and encourage the creation of new ones. In 2015, the number of qualified SVOs was 5 in the North region; 13 in the Northeast; 20 in the Southeast; 5 in the South; and 9 in the Centre-West [42].

The information systems used allow the registration of the ICD referring to *E. multilocularis*, which is not present in Brazil and, coupled with the lack of a specific code for the registration of events involving *E. vogeli* and *E. oligarthrus*, results in gaps in the understanding of the epidemiology and spatialization of cases in the country.

There are several limitations to our knowledge of CE-related deaths in Brazil and consequently this study. With the change of disease coding and the integration of echinococcosis with other pathologies, there is a lack of record specificity. Underreporting of deaths due to CE, either due to the disease not being universally notifiable, or because of insufficient diagnostic facilities, also highlights a margin of error in the reported deaths and consequently our results.

## 5. Conclusions

Echinococcosis is a neglected tropical disease that causes considerable burden to the health systems of affected countries. Here, we described the registered deaths from the disease in Brazil, as documented in two mortality recording systems, between 1995 and 2016. This article shows that the disease does not receive the necessary attention from public authorities in the adoption of prevention and control measures, and that further studies are needed to identify the real causes of deaths from CE registered in information systems, as well as the etiological agent involved in the deaths.

The disease can be considered endemic in Brazil and the records of deaths are distributed in all regions. The difficulty of diagnosis and verification of death may contribute to the low number of deaths attributed to CE in the evaluated information systems. Elucidating the true burden of disease, in both humans and zoonotic species, would enable a more thorough understanding of the disease transmission dynamics and risk factors for disease transmission. A surveillance system extending beyond endemic areas, increased diagnostic capacity, and more accurate measurements of the disease burden, including costs to the public health systems, are still an urgent necessity in Brazil.

## Figures and Tables

**Table 1:** Distribution of Mortality Rate in HIS, by FU, Brazil, 1995–2016.

Federal Unit (FU)	No. Admissions	No. Deaths	Mortality Rate (%)	Mortality per 100,000 ppl
Rondônia	139	0	0.00	0.00
Acre	78	2	2.56	0.36
Amazonas	45	0	0.00	0.00
Roraima	9	0	0.00	0.00
Pará	380	6	1.58	0.12
Amapá	8	0	0.00	0.00
Tocantins	20	0	0.00	0.00
Maranhão	1007	1	0.10	0.02
Piauí	389	2	0.51	0.08
Ceará	234	4	1.71	0.06
Rio Grande do Norte	136	4	2.94	0.14
Paraíba	99	1	1.01	0.03
Pernambuco	274	11	4.01	0.14
Alagoas	544	16	2.94	0.57
Sergipe	12	0	0.00	0.00
Bahia	1492	59	3.95	0.45
Minas Gerais	260	7	2.69	0.04
Espírito Santo	165	2	1.21	0.08
Rio de Janeiro	303	19	6.27	0.13
São Paulo	475	22	4.63	0.07
Paraná	207	4	1.93	0.05
Santa Catarina	244	6	2.46	0.11
Rio Grande do Sul	854	17	1.99	0.17
Mato Grosso do Sul	13	1	7.69	0.05
Mato Grosso	436	1	0.23	0.04
Goiás	63	0	0.00	0.00
Distrito Federal	69	0	0.00	0.00

**Table 2:** Distribution of deaths and Mortality rate per 100,000 inhabitants registered in MIS, by FU and International Classification of Disease-10, Brazil, 1999–2016.

FU of Residence	B67.0	B67.1	B67.2	B67.3	B67.4	B67.6	B67.8	B67.9	Total	%	Mortality Rate
Rio Grande do Sul	8	7	0	2	3	1	13	17	51	45.53	0.48
Acre	0	0	0	0	0	0	1	13	14	12.39	1.85
Pará	0	0	0	0	0	0	1	9	10	8.85	0.13
São Paulo	1	1	0	0	0	0	1	5	8	7.08	0.02
Rondônia	0	0	0	0	0	0	2	1	3	2.65	0.19
Piauí	0	0	1	0	0	0	1	1	3	2.65	0.10
Alagoas	0	0	2	0	0	0	1	0	3	2.65	0.10
Bahia	0	1	0	0	0	0	2	0	3	2.65	0.02
Tocantins	0	0	0	0	0	0	0	2	2	1.77	0.14
Ceará	0	0	0	0	0	0	2	0	2	1.77	0.02
Santa Catarina	1	0	0	0	0	0	1	0	2	1.77	0.03
Mato Grosso do Sul	0	0	0	0	0	0	2	0	2	1.77	0.08
Goiás	0	0	0	1	0	0	0	1	2	1.77	0.03
Amapá	0	0	0	0	0	0	0	1	1	0.88	0.14
Rio Grande do Norte	0	0	0	0	0	0	1	0	1	0.88	0.03
Minas Gerais	0	0	0	0	0	0	0	1	1	0.88	0.01
Rio de Janeiro	0	0	0	0	0	0	0	1	1	0.88	0.01
Paraná	0	0	0	0	0	0	0	1	1	0.88	0.01
Mato Grosso	0	0	0	0	0	0	1	0	1	0.88	0.03
Distrito Federal	0	1	0	0	0	0	0	0	1	0.88	0.04
Total	10	10	3	3	3	1	29	53	112	100	0.06
% Total/CID	8.85	9.73	2.65	2.65	2.65	0.88	25.66	46.9	100		

Legend: B67.0 Liver infection by *Echinococcus granulosus*; B67.1 Pulmonary infection by *Echinococcus granulosus*; B67.2 Bone infection by *Echinococcus granulosus*; B67.3 *Echinococcus granulosus*, other and multiple site infections; B67.6 *Echinococcus multilocularis* infections, other and multiple sites; B67.8 Liver infection, unspecified, by *Echinococcus*; B67.9 *Echinococcus* infections, other, and unspecified.

**Table 3:** Distribution of deaths recorded in the MIS, by etiological agent and location of the cyst in human organs/tissues, Brazil, 1999–2016.

Etiological Agent	Cyst Location	Number	%
*E. granulosus*	Hepatic	10	8.85
	Pulmonary	11	9.73
	Bone	3	2.65
	Other *	3	2.65
	Not specified	3	2.65
*E. multilocularis*	Hepatic	1	0.88
*Echinococcus* spp.	Hepatic	29	25.66
	Not specified	53	46.9
Total		113	−100

* Pertains to other sites of cyst location other than those explicitly specified.

## Data Availability

The data presented in this study are openly available on the Brazilian Ministry of Health website: To access the HIS: https://datasus.saude.gov.br/acesso-a-informacao/producao-hospitalar-sih-sus/; to access the MIS: https://datasus.saude.gov.br/mortalidade-desde-1996-pela-cid-10.

## Abbreviations

**Name**	**Abbreviations**
Hospital information system	HIS
Mortality information system	MIS
Echinococcosis	CE
Alveolar echinococcosis	AE
Polycystic neotropical echinococcosis	PNS
Unicystic echinococcosis	UES
World Health Organisation	WHO
Hospital mortality rate	HMR
lethality rate	LR
standardized mortality rate	SMR
federal unit	FU
